# Economic burden of opioid misuse focused on direct medical costs

**DOI:** 10.3389/fphar.2022.928890

**Published:** 2022-10-14

**Authors:** Miryoung Kim, Siin Kim, Hae Sun Suh

**Affiliations:** ^1^ College of Pharmacy, Pusan National University, Busan, South Korea; ^2^ College of Pharmacy, Kyung Hee University, Seoul, South Korea; ^3^ Department of Regulatory Science, Graduate School, Kyung Hee University, Seoul, South Korea

**Keywords:** opioid, opioid misuse, opioid abuse, economic burden of disease, burden of disease (BOD)

## Abstract

**Background:** Since their development, synthetic opioids have been used to control pain. With increased opioid use, problematic opioid prescription has also increased, resulting in a growing economic burden. However, there is a paucity of research studies on the economic burden of prescription opioid misuse in Asia, especially South Korea.

**Objectives:** To estimate the incremental economic burden of prescription opioid misuse for the South Korean population.

**Methods:** The National Health Insurance Service-National Sample Cohort database, covering 2% of the South Korean population between 2010 and 2015, was analyzed. Outpatients aged 18 or older who took one or more prescription opioids were selected. Based on their opioid prescription patterns, patients were classified into opioid misuse and non-misuse groups. The direct medical costs per person per year (PPPY) and the incremental economic burden of the opioid misuse group were explored using an exponential conditional model with a suitable distribution and log link function. All analyses were performed using SAS® Enterprise Guide version 9.4, and *p* < 0.05 was considered statistically significant.

**Results:** The number of patients who had ≥1 opioid prescription was 345,020 including 84,648 (24.53%) in the opioid misuse group and 260,372 (75.47%) in the non-misuse group. The adjusted mean direct medical costs PPPY were estimated to be USD 401 for the opioid misuse group, which is 1.49 times significantly higher than that for the non-misuse group (*p* < 0.0001). The incremental economic burden of the opioid misuse group in the South Korean population was estimated to be approximately USD 0.52 billion for the period 2010–2015.

**Conclusion:** Prescription opioid misuse was significantly associated with the increased economic burden. Along with proper policies for using opioids, it is necessary to monitor opioid prescription patterns to prevent opioid misuse and reduce the related economic burden.

## Introduction

For decades, opioids have been used all over the world to manage pain. Since the identification of various opioid receptors led to the development of synthetic opioids, their prescription has steadily increased (Cherny, 1996; Sullivan et al., 2008; Trescot et al., 2008). Opioids are effective analgesic drugs that do not have a ceiling effect—a pharmacological phenomenon in which a drug’s effect reaches a plateau; however, this attribute can lead to adverse effects including sedation, nausea, vomiting, and respiratory depression (Trescot et al., 2008). In addition, with the increased use of opioids in pain management, problematic prescription opioid incidents such as overdose, dependence, and addiction have also increased (Vowles et al., 2015). Furthermore, in 2014, the reported total number of non-medical prescription opioid users was 10.3 million (Abuse and Administration, 2014). In terms of non-medical prescription opioid use, the rate of heroin use increased by approximately 140% between 2002 and 2004 and the period of 2011–2013 (Compton et al., 2016). The rate of death from prescription opioid overdose approximately quadrupled between 2000 and 2014. From 2019 to 2020, the rate increased by over 16% (CDC National Center for Health Statistics, 2021).

Prescription opioid misuse and abuse create not only public health issues, including overdose and death, but also a growing economic burden globally (Meyer et al., 2014). Birnbaum et al. (2006) and Birnbaum et al. (2011) revealed that healthcare costs related to prescription opioid abuse in the US rose from $2.6 billion in 2001 to $25.0 billion in 2007. While the authors investigated the overall costs of opioid abuse in the US, some studies analyzed the average costs per person adjusting for differences in patient demographics. After matching the opioid abuse group with the non-abuse group, the average cost for each group was calculated (White et al., 2005). However, they did not appropriately manage the skewness of cost data, which could lead to incorrect average cost estimates, nor did they examine the relationship between costs and patient characteristics.

In South Korea, health insurance is a universal public insurance system covering over 97% of the entire population (Seong et al., 2017; Kwon, 2009). Owing to this health insurance system, access to healthcare in South Korea is easier than in other Organization for Economic Co-operation and Development (OECD) member nations. The number of doctor consultations per person in Korea is the highest among OECD member nations (14.7 in Korea vs. 5.9 in OECD nations on average in 2020) (OECD.Stat, 2022). Access to opioids is no exception. In Korea, all forms of opioids can only be obtained *via* prescription under the Narcotics Control Act (Korean Law Information Center, 2022). Despite strong regulations, the rate of opioid prescription has increased. According to South Korean studies on prescription patterns, the opioid prescription rate per 1,000 persons almost doubled from 347.5 in 2009 to 531.3 in 2019 (Cho et al., 2021). Specifically, cases of opioid prescription have soared since 2010 (Kim et al., 2022). However, there is a paucity of evidence on the economic burden of opioid prescription use in Asia, including South Korea. Moreover, to the best of our knowledge, no studies have been conducted to estimate the size of opioid abuse and its associated economic burden in Asia.

Therefore, using econometric models, our study estimated the total economic burden of prescription opioid misuse adjusting for non-cancer patients considering the skewness of cost data and seeks to fill the evidence gap in the Asian population. We also estimated the incremental costs related to opioid misuse in comparison with non-misuse in South Korea.

## Materials and methods

### Data source and study population

**FIGURE 1:**
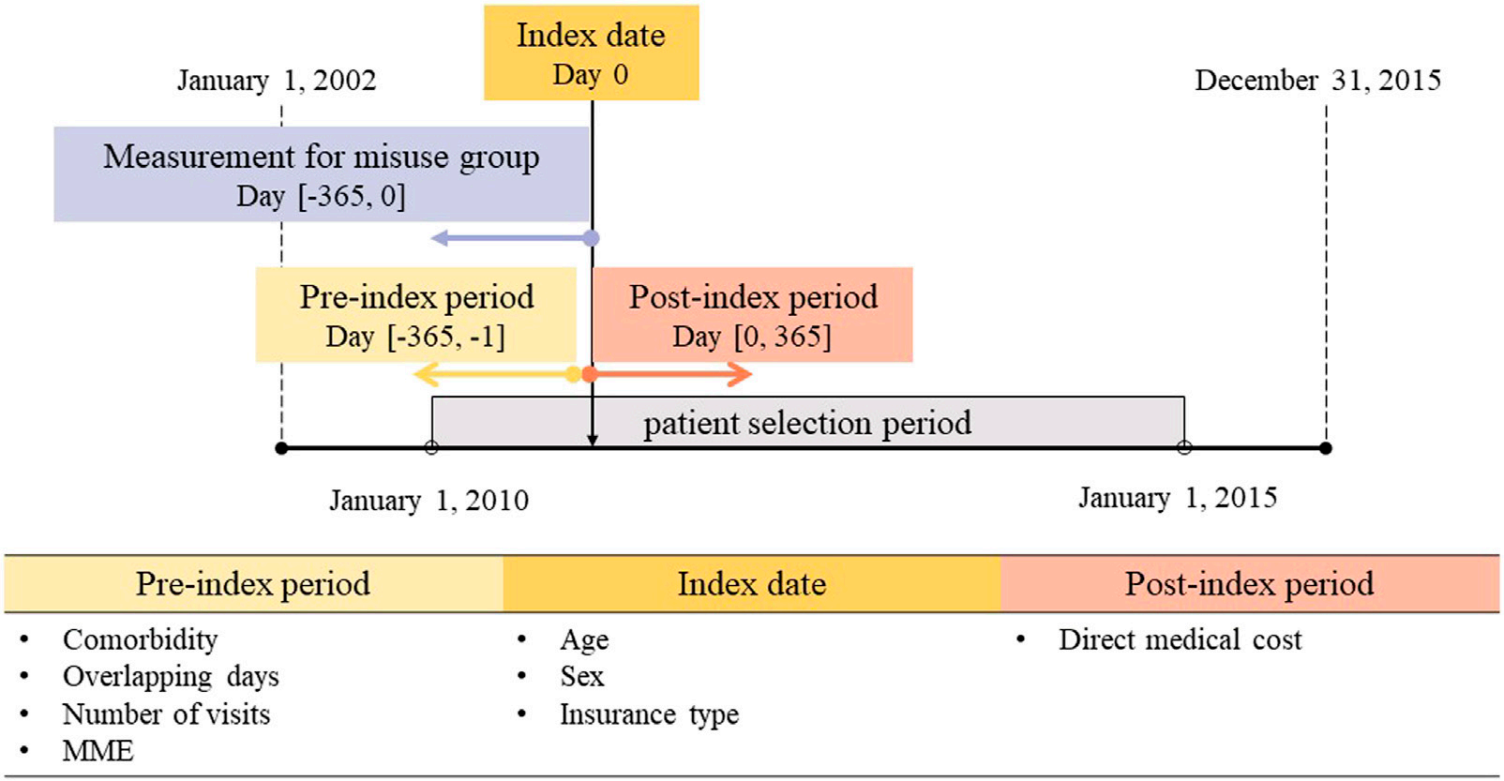
Study scheme. MME, morphine milligram equivalent.

Data were drawn from the National Health Insurance Service-National Sample Cohort (NHIS-NSC) database version 2.0 from 2002 to 2015. The NHIS-NSC is a representative database that covers 2.2% of the South Korean population (Lee et al., 2017). The study period ranged from 1 January 2002 to 31 December 2015 (Figure 1). The eligible study population comprised outpatients aged 18 years or older who took one or more prescription opioids between 1 January 2010 and 1 January 2015. Patients with cancer diagnoses who had ICD-10 codes C00–C97, except C44 [other malignant neoplasms of the skin], were excluded from our study during the entire study period. Prescription opioids were defined based on the US Drug Enforcement Administration’s Schedule II–IV opioids and the Korean guideline’s category; they can be enumerated as follows (Sullivan et al., 2008; Kim et al., 2017; Carey et al., 2018; Kim et al., 2021): buprenorphine, codeine, dihydrocodeine, fentanyl, hydromorphone, hydrocodone, meperidine, morphine, oxycodone, pentazocine, and tramadol.

The eligible study population was classified into opioid misuse and non-misuse groups based on the patients’ opioid prescription patterns. Since the definition of opioid misuse is not straightforward using claims data, the most widely used indicators for opioid misuse are opioid shopping and overlapping prescriptions (Sullivan et al., 2008; Yang et al., 2015; Kim et al., 2017; Carey et al., 2018; Kim et al., 2021). Yang et al. (2015) found that opioid prescription patterns—more than four different pharmacies within 90 days—was significantly associated with an increased risk of overdose. The index date was defined as the first date of opioid prescription between 1 January 2010 and 1 January 2015. The opioid misuse group was defined as outpatients who met at least one of the following criteria prior to the index date within 1 year: 1) they visited more than four different hospitals to obtain prescription opioids during any 90-day period (Sullivan, 2013; Yang et al., 2015; Carey et al., 2018; Kim et al., 2021) and 2) they had prescriptions with more than 25% overlapping days with the next prescription for opioids containing the same active ingredient (Yang et al., 2015; Kim et al., 2021). The non-misuse group was defined as the cohort of opioid users that did not fulfill the aforementioned criteria.

### Covariates and outcome measures

The baseline characteristics included sex, age, insurance type, the Charlson comorbidity index (CCI) score, the mean overlapping days with the next prescription, the mean number of opioid-related visits by outpatients, and the morphine milligram equivalent (MME). MME is a measurement that converts an opioid dose to its equivalent morphine dose. The CCI score, mean overlapping days, and mean number of visits were evaluated in the pre-index period 1 year before the index date, whereas the others were assessed on the index date. In South Korea, there are two types of health insurance, and the ratio of the co-payment depends on patients’ income levels: the National Health Insurance (NHI), in which the co-payment ranges from 30% to 60%, and the Medical Aid Program, available for people with low incomes, in which the co-payment ranges from 10% to 30% (Chun et al., 2009). The major diagnoses for the misuse group were examined according to the total medical cost and frequency.

We explored the direct medical costs per person per year (PPPY) as the outcomes. These were assessed by analyzing opioid prescriptions during 1 year after the index date. To avoid overestimation of outcomes, prescriptions accompanied by surgery were excluded. This was because opioids are essential during surgery for both the opioid misuse and non-misuse groups. For both groups, the direct medical cost was comprised of outpatient costs and hospitalization costs. Outpatient costs were the sum of the outpatient visit costs and the outpatient drug costs. Outpatient visit costs included the doctors’ visiting costs and the cost of drugs that patients were administered at the institutions. Outpatient drug costs included the total opioid and non-opioid costs charged at pharmacies. Hospitalization costs included all expenses for services provided during hospitalization, such as room rates, medical imaging fee, laboratory test fee, nursing fee, and drug costs. All costs were the incurred costs paid by the NHIS and patients.

Mean direct medical costs PPPY were estimated for each group and were used to calculate the incremental costs PPPY. The incremental economic burden of the opioid misuse group in the South Korean population during the period 2010–2015 was calculated using the following equation:

The incremental economic burden of opioid misuse in the South Korean population in 2010 –2015

$$\begin{array}{ll} = & (The\;adjusted\;mean\;incremental\;cost\;per\;person\;per\;year\;of\;the\;opioid\;misuse\;group)\\ & \times (the\;prevalence\;of\;opioid\;misuse\;in\;the\;NHIS–NSC\;population\;in\;2010\;–2015)\\ & \times (the\;average\;total\;number\;of\;South\;Korean\;population\;in\;2010\;–2015). \end{array}$$ (1)

The prevalence of opioid misuse was calculated by dividing the number of patients in the misuse group by the total number of the NHIS-NSC population from 2010 to 2015. Data on the South Korean population were obtained from the Korean Statistical Information Service (Korean Statistical Information Service, 2021).

### Statistical analysis

To compare the baseline characteristics between the opioid misuse and non-misuse groups, the chi-squared test was used for categorical variables, and the student’s t-test and Wilcoxon rank sum test were used to for continuous variables. To consider the property of cost data characterized by the positively skewed distribution, we first confirmed the normality using a histogram and the Kolmogorov–Smirnov test. The mean cost was estimated using an exponential conditional model (ECM), which has the structure of a generalized linear model (GLM) (Manning and Mullahy, 2001). The GLM can estimate the cost with a suitable distribution and link function and can identify the association between covariates and cost. The suitable distributions for cost were selected based on a modified Park test. The Akaike information criterion (AIC) was used to compare regression models in terms of goodness of fit (Manning and Mullahy, 2001; Barber and Thompson, 2004; Nixon and Thompson, 2004). In the case of including a zero value, we selected the GLM with the Tweedie distribution and log link function (Lord et al., 2005;Kurz, 2017). When modeling the GLM, the following covariates were included: age, sex, insurance type, CCI score, comorbid diseases, and MME.

Costs were recorded in Korean won (KRW), which was converted to United States dollar (USD) using the average 2020 exchange rate (1 USD = 1,180.27 KRW). All analyses were performed using SAS^®^ Enterprise Guide version 9.4, and the statistical significance was set at *p* < 0.05. The Institutional Review Board of Pusan National University granted an exemption from an Institutional Review Board review for this study (PNU IRB/2020_76_HR).

## Results

### Baseline characteristics

Of the NHIS-NSC population from 2010 to 2015, the number of patients who had one or more opioid prescriptions was estimated to be 345,020, of which 84,648 (24.53%) belonged to the opioid misuse group and 260,372 (75.47%) to the non-misuse group (Table 1). Among the opioid misuse group, 60.43% were female, and the mean age was 54.03 ± 15.30 (mean ± standard deviation). The proportion of Medical Aid recipients in the opioid misuse group was higher than that in the non-misuse group (5.45% vs. 2.57%). The CCI score was significantly higher in the opioid misuse group than in the non-misuse group (0.67 ± 0.89 vs. 0.42 ± 0.72, *p* < 0.0001). The mean value of overlapping days in the former group was higher than that in the latter (0.81 ± 1.64 vs. 0.01 ± 0.10 days, *p* < 0.0001). The opioid misuse group visited clinics or hospitals for opioid prescriptions on an average of 26.01 ± 39.92 times per year and consumed a significantly larger amount of MME (27.85 ± 112.68 vs. 16.63 ± 63.00 mg, *p* < 0.0001) than the non-misuse group during this period. The medical costs of dorsalgia accounted for the highest proportion of the total medical costs related to opioids for the misuse group, at 7.75%, followed by arthrosis of the knee (7.5%) and other spondylopathies (5.97%). The list of the diagnoses according to the total medical cost and frequency is presented in the Supplementary Appendix SA1.

**TABLE 1:** Baseline characteristics of the study population.

Characteristic	Misuse (*n* = 84,648) No. (%) or mean (SD)	Non-misuse (*n* = 260,372) No. (%) or mean (SD)	*p*-value^a^
Female	51,156 (60.43)	141,457 (54.33)	<0.0001
Age, years	54.03 (15.30)	44.49 (15.38)	<0.0001
Age group			<0.0001
18–29 years	5,718 (6.76)	49,025 (18.83)	
30–39 years	10,044 (11.87)	55,694 (21.39)	
40–49 years	15,838 (18.71)	59,362 (22.80)	
50–59 years	20,839 (24.62)	51,807 (19.90)	
60–69 years	17,132 (20.24)	26,544 (10.19)	
70–79 years	12,250 (14.47)	14,196 (5.45)	
≥80 years	2,827 (3.34)	3,744 (1.44)	
Insurance			<0.0001
NHI program	80,034 (94.55)	253,689 (97.43)	
Medical Aid program	4,614 (5.45)	6,683 (2.57)	
CCI score^b^	0.67 (0.89)	0.42 (0.72)	<0.0001
Overlapping days^b^	0.81 (1.64)	0.01 (0.10)	<0.0001
No. of outpatient visits^b^	26.01 (39.92)	5.19 (8.34)	<0.0001
MME, mg	27.85 (112.68)	16.63 (63.00)	<0.0001
MME group			<0.0001
20 mg >	51,232 (60.52)	235,478 (90.44)	
20–49 mg	28,581 (33.76)	23,157 (8.89)	
50–89 mg	3,090 (3.65)	663 (0.25)	
≥90 mg	1,745 (2.06)	1,074 (0.41)	

SD, standard deviation; CCI, Charlson comorbidity index; MME, morphine milligram equivalent; NHI, National Health Insurance.

^a^ The chi-squared test was used for categorical variable analysis, and both the *t*-test and Wilcoxon rank sum test were used for continuous variable analysis.

^b^ These were evaluated in the pre-index period.

### Direct medical costs and the incremental burden of the opioid misuse group

The distribution of the observed direct medical costs was severely skewed to the right with a long tail. The arithmetic mean of the direct medical costs PPPY (USD 155.48) was substantially greater than its medians (USD 58.87). Based on the Kolmogorov–Smirnov test results, the null hypothesis that the data were sampled from a normal distribution was rejected (*p* < 0.0001). As seen in Table 2, the unadjusted mean direct medical costs PPPY, not controlling for any covariates, were USD 256.48 and USD 122.65 in the misuse and non-misuse groups, respectively. A large standard deviation was observed for both groups (691.98 vs. 387.27).

**TABLE 2:** Observed mean direct medical cost and adjusted mean direct medical cost by modeling^a^.

		Misuse		Non-misuse		
	AIC	Adjustedmean cost (USD)	SE	Adjustedmean cost (USD)	SE	Δ mean
Observed cost	—	256.48^b^	691.98^c^	122.65^b^	387.27^c^	+133.83
GLM, inverse Gaussiandistribution	3919294	401.18	7.33	268.81	4.98	+132.37
GLM, gamma distribution	4072729	402.30	3.58	263.67	2.43	+138.63
GLM, normal distribution	6128952	386.49	25.72	241.51	18.76	+144.98

AIC, Akaike information criterion; SE, standard error; GLM, generalized linear model with the log link function.

^a^ We estimated the mean direct medical cost per person per year under following control covariates: sex, age, insurance type, the Charlson comorbidity index score, comorbid diseases, and morphine milligram equivalent.

^b^ Arithmetic mean cost.

^c^ Standard deviation.

The modified Park test results suggested that the distribution of the direct medical costs PPPY lay somewhere between the gamma and the inverse Gaussian distribution (variance function powers *λ* = 2.21). The direct medical costs PPPY were best fitted by the GLM with the inverse Gaussian distribution and log link function because the AIC was the lowest. The adjusted mean direct medical costs PPPY with the GLM were estimated as USD 401.18 ± 7.33 for the opioid misuse group. The adjusted mean incremental cost PPPY of this group relative to the non-misuse group was USD 132.37 (Table 2).

**FIGURE 2:**
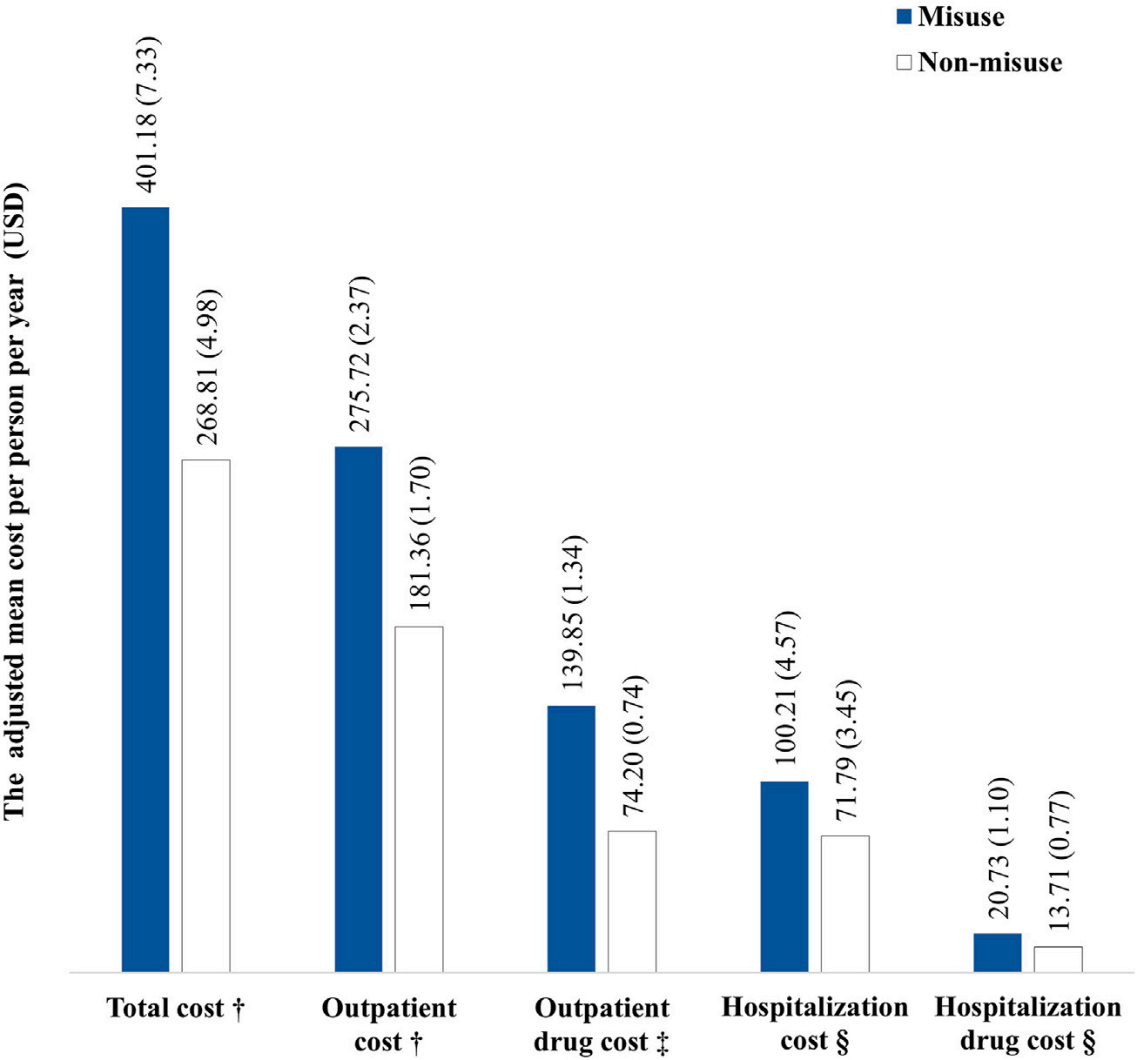
Adjusted mean costs per person per year in the misuse and non-misuse groups. ^†^The generalized linear model (GLM) with the inverse Gaussian distribution and log link function. ^‡^The GLM with the gamma distribution and log link function. ^§^The GLM with the Tweedie distribution and log link function. Note: the total cost was the sum of the outpatient and hospitalization costs. Outpatient costs were the sum of the outpatient visits and drug costs. Hospitalization costs included room rates, medical imaging fee, laboratory test fee, nursing fee, and drug costs.

Using the GLM with the inverse Gaussian distribution and log link function that had the lowest AIC, all components of the total direct medical costs were found to be higher in the opioid misuse group than in the non-misuse group (Figure 2). The outpatient costs PPPY were USD 275.72 ± 2.37 and USD 181.36 ± 1.70 in the misuse and non-misuse groups, respectively. Of the outpatient costs, the outpatient drug costs PPPY were USD 139.85 ± 1.34 and USD 74.20 ± 0.74 in the misuse and non-misuse groups, respectively. The outpatient drug costs were best fitted by the GLM with the gamma distribution and log link function.

A total of 2.97% of the overall study population had been hospitalized more than once (4.28% and 2.54% for the misuse and non-misuse groups, respectively). The hospitalization costs PPPY were USD 100.21 ± 4.57 and USD 71.79 ± 3.45 in the misuse and non-misuse groups, respectively. The drug costs PPPY incurred during hospitalization were USD 20.73 ± 1.10 and USD 13.71 ± 0.77 for the misuse and non-misuse groups, respectively. Both were estimated using the GLM with the Tweedie distribution and log link function.

The prevalence of opioid misuse in the NHIS-NSC population between 2010 and 2015 was calculated to be 7.76%. The total number of patients in the NHIS-NSC population during the period was 1,091,257, and the number of patients in the misuse group was 84,648. During the same period, the average population of South Korea was estimated to be 50,313,517 (Korean Statistical Information Service, 2021). The total economic burden of the opioid misuse group was estimated to be approximately USD 1.57 billion, and the incremental economic burden of the opioid misuse group compared to the non-misuse group was 0.52 billion USD.

### Association between total direct medical cost and patient characteristics

As previously shown, the mean direct medical costs were estimated using the GLM with the inverse Gaussian distribution and log link function since it showed the lowest AIC. The coefficients of covariates estimating the mean direct medical costs are reported in Table 3. Almost all the coefficients were significantly associated with the direct medical costs (*p* < 0.0001), except for chronic obstructive pulmonary disease (*p* = 0.7288), dementia (*p* = 0.6141), myocardial infarction (*p* = 0.9721), and paraplegia (*p* = 0.8058). The mean direct medical costs for the misuse group were 1.49 times significantly higher than those for the non-misuse group. Those for males were 1.05 times significantly higher than those for females. Moreover, such costs were 1.48 times significantly higher for Medical Aid patients than for NHI patients. Additionally, older age, higher CCI scores, and higher MME were significantly associated with increased costs. The following comorbidities were significantly associated with increased costs: back pain, cerebrovascular disease, congestive heart failure, diabetes, liver disease, peptic ulcer, peripheral vascular disease, and rheumatic disease.

**TABLE 3:** Association between the direct medical cost and patient characteristics measured by GLM with the inverse Gaussian distribution and log link function.

Covariate	Estimate	SE	95% confidence limit		*p*-value
Intercept	4.2754	0.0056	4.2644	4.2865	<0.0001
Group (ref. non-misuse)					
Misuse	0.4004	0.0073	0.3860	0.4147	<0.0001
Sex (ref. female)					
Male	0.0534	0.0049	0.0438	0.0631	<0.0001
Age (ref. 18–29 Y)					
30–39 Y	0.1304	0.0069	0.1169	0.1439	<0.0001
40–49 Y	0.2770	0.0070	0.2633	0.2908	<0.0001
50–59 Y	0.5299	0.0078	0.5146	0.5453	<0.0001
60–69 Y	0.6644	0.0103	0.6442	0.6846	<0.0001
70–79 Y	0.8890	0.0143	0.8609	0.9171	<0.0001
≥80 Y	1.0790	0.0292	1.0218	1.1363	<0.0001
Insurance (ref. NHI)					
Medical Aid	0.3904	0.0184	0.3543	0.4264	<0.0001
CCI score (ref. CCI=0)					
CCI = 1	0.1238	0.0063	0.1114	0.1362	<0.0001
CCI = 2	0.3285	0.0130	0.3030	0.3540	<0.0001
CCI = 3	0.5780	0.0221	0.5347	0.6214	<0.0001
MME (ref. 20 mg>)					
20–49 mg	0.0781	0.0076	0.0631	0.0931	<0.0001
50–89 mg	0.2445	0.0316	0.1826	0.3065	<0.0001
≥90 mg	0.8375	0.0493	0.7408	0.9342	<0.0001
Comorbid disease dummy					
Back pain	0.1428	0.0080	0.1272	0.1585	<0.0001
CERE	0.a1384	0.0318	0.0760	0.2007	<0.0001
CHF	0.1965	0.0465	0.1053	0.2878	<0.0001
COPD	0.0032	0.0091	−0.0147	0.0211	0.7288
Dementia	0.1643	0.3260	−0.4745	0.8032	0.6141
DM	0.1198	0.0208	0.0789	0.1606	<0.0001
Liver	0.1548	0.0147	0.1261	0.1836	<0.0001
MI	0.0030	0.0857	−0.1649	0.1709	0.9721
Paraplegia	−0.0282	0.1148	−0.2533	0.1969	0.8058
PUD	0.0344	0.0095	0.0158	0.0530	0.0003
PVD	−0.0689	0.0300	−0.1277	−0.0101	0.0216
RM	0.2859	0.0301	0.2270	0.3448	<0.0001
Scale	0.1269	0.0002	0.1266	0.1272	

Number of observations = 345,020. Dependent variable = the mean direct medical cost. GLM, generalized linear model; CCI, Charlson comorbidity index; MME, morphine milligram equivalent; NHI, National Health Insurance; CERE, cerebrovascular disease; CHF, congestive heart failure; COPD, chronic pulmonary disease; DM, diabetes; Liver, liver disease; MI, myocardial infarction; PUD, peptic ulcer disease; PVD, peripheral vascular disease; RM, rheumatic disease.

## Discussion

This study estimated the incremental direct medical burden of opioid misuse. We estimated the mean direct medical costs PPPY in the opioid misuse and non-misuse groups after adjustments based on the econometric model. The former group incurred significantly higher direct medical costs than the latter group. The estimated mean direct medical costs PPPY for the misuse group were approximately 1.5 times higher than those for the non-misuse group.

The estimated prevalence of opioid misuse during the period 2010–2015 was 7.76%. We were not able to compare this estimate with others since none of the previous studies reported the prevalence of opioid misuse in South Korea. Studies conducted in the US have determined opioid misuse rates between 2.0% and 56.3% (Vowles et al., 2015). Although differences between countries may exist, the estimated prevalence of opioid misuse in this study is likely to be reasonable. Considering the estimated prevalence and the average population of South Korea, the economic burden of opioid misuse for the South Korean population was estimated to be approximately USD 1.57 billion in 2010–2015. The incremental economic burden of opioid misuse was estimated to be approximately USD 0.52 billion during the same period.

According to the National Health Insurance Statistical Yearbook in South Korea in 2015, the NHIS financing expenditure was USD 40.81 billion. According to the results of studies on disease burdens in South Korea in 2015, the direct medical costs were estimated to be approximately USD 2.65 billion for stroke, USD 0.32 billion for hepatitis B, and USD 0.15 billion for depression (Baik et al., 2020; Cha, 2018; Chang et al., 2012). In the current study, the incremental economic burden of opioid misuse per year was estimated to be approximately USD 86 million, which is noteworthy compared to other disease burdens. The annual opioid prescriptions in South Korea increased continuously from 2009 to 2019 (Cho et al., 2021). According to this trend, the economic burden of opioid prescriptions is expected to increase, especially in the misuse group, implying that the opportunity cost of opioid misuse is expected to steadily increase as well.

Previous studies defined opioid misuse from healthcare claims data by using specific patterns of opioid prescriptions. The most widely used indicators for opioid misuse are opioid shopping and overlapping prescriptions. Yang et al. (2015) revealed that an opioid misuse defined by both indicators (i.e., pharmacy shopping and overlapping prescriptions) was significantly associated with an increased risk of overdose. In this study, we used both indicators to differentiate between patients with and without opioid misuse and compared these two groups to assess the incremental economic burden of opioid misuse.

Rice et al. (2012) determined that the probability of opioid abuse in the US was associated with the number of opioids, antipsychotics, or hypnotics as concomitant medication and mental illness as comorbid conditions. Patient characteristics affecting the probability of opioid misuse were generally similar between the US and South Korea (Noh et al., 2022). In contrast with the US (odds ratio (OR) = 0.59 for older people, 60–64 years old), the risk of an inappropriate prescription of opioids in South Korea was higher in females and older people (OR = 2.12 for older people, ≥ 65 year old). Notably, polypharmacy (≥10 medications) was significantly associated with the inappropriate prescription of opioids in South Korea (OR = 18.5) (Noh et al., 2022). Kim et al. (2014) revealed that over 80% of people of age 65 years and above had polypharmacy in South Korea. In other words, polypharmacy in older people in South Korea may be associated with the probability of opioid misuse.

South Korea has various programs, regulations, and policies for preventing opioid misuse. The Korean Association Against Drug Abuse is the nation’s only private organization that carries out comprehensive projects for the prevention of drug abuse such as the construction of community networks and the undertaking of research activities and educating high-risk groups (Korean Association Against Drug Abuse, 2022). Since 2018, all individuals who handle narcotics in South Korea have been mandated to legally report the narcotic details (e.g., product name, the quantity consumed, stock, and serial number) and patients’ information to the web-based Narcotics Information Management System (NIMS): exporter and importer, manufacturer, wholesaler, pharmacist, healthcare provider, and researcher. The NIMS can contribute to the identification and management of opioid prescription patterns to prevent its misuse. In addition, the “Network System to Prevent Doctor-shopping for Narcotics” was implemented by the NIMS in 2021 to verify previous narcotic prescriptions and evaluate the risk of abuse. The healthcare provider can access the patient’s narcotic history from the NIMS database and examine the previous narcotic prescriptions. This system has been assessed to be a cost-effective method for preventing opioid abuse (Kim et al., 2021). We recommend that the NIMS should be actively utilized to evaluate prescription opioid patterns and prevent opioid misuse. For this, we suggest an additional policy, the compulsory assessment of the patient’s opioid history, before prescribing opioids. The compulsory assessment can help reduce the risk of misuse and provide appropriate treatment for patients who need pain control. Moreover, a decrease in the healthcare expenditure is expected.

By matching differences in patient characteristics between patients with and without opioid abuse, White et al. (2005) demonstrated that the direct medical costs of opioid abuse were approximately eight times higher than those of opioid non-abuse. They calculated the average costs per person, regardless of the nature of the cost data in terms of distribution. Since most cost data are right-skewed, it may violate the assumptions of a normal distribution required to calculate the average costs, resulting in inaccurate estimates. Skewed data are the main issue in statistical models in healthcare costs which tend to be skewed to the right. This occurs because a large number of costs cluster around a lower range of values (left-hand side), whereas a few high-cost values are present in the tail (right-hand side) (Manning and Mullahy, 2001; Thompson and Barber, 2000). In other words, the right-skewed distribution cost means that the number of patients with low expenditure is high; those with high expenditure are relatively rare. Since the mean is typically greater than the median in this case, we need to be cautious when estimating the mean to obtain unbiased and precise estimates. To do so, we used the ECM, which can estimate the unbiased and precise mean even with non-symmetric data. Although the linear regression model with logarithmic transformation can make the skewed data symmetric, the transformation creates the problem of retransformation of estimates back to an economically meaningful scale. In contrast, ECM assumes a nonlinear relationship for the cost regression; therefore, it allows avoiding retransformation. In summary, to consider the distribution of costs, we used the GLM, which has a distribution family function for the dependent variable and the link function that describes the relationship with covariates. This flexibility allows for the estimation of non-normal distributions (Afifi et al., 2007; Barber and Thompson, 2004; Dodd et al., 2006). In our direct medical costs data, the variance function power was 2.21, and the inverse Gaussian distribution showed the lowest AIC.

The present study has several notable strengths. First, we estimated an incremental economic burden for the opioid misuse group in Asia. This is expected to be useful in forecasting the incremental economic burden of such groups in Asian countries, where the rate of opioid misuse is different from that of Western countries. Health policymakers would be able to understand the magnitude of the burden related to opioid misuse. Moreover, the results provide evidence for comparison with the burden of other diseases so that health policymakers would be able to prioritize certain policies over others. Second, we estimated the economic burden by using real-world data representing the entire population of South Korea. Third, we considered a skewed distribution of cost data and estimated the economic burden more accurately by adjusting for patient characteristics.

There are several limitations that should be acknowledged. First, it was difficult to clearly define opioid misuse. Although some characteristics, such as overdose and addictions, are distinguishable based on their diagnosis codes, we were not able to use these codes because these were masked in the NHIS-NSC database to protect personal information. However, we overcame this limitation by classifying opioid users into the opioid misuse and non-misuse groups based on their opioid prescription patterns related to overdose or shopping. To classify opioid prescription misuse patterns, we applied an operational definition based on previous studies (Sullivan, 2013; Yang et al., 2015; Carey et al., 2018; Kim et al., 2021). Second, we were unable to determine the exact prevalence of opioid misuse in the entire South Korean population. Instead, we used the estimated prevalence in the NHIS-NSC population because it contains representative data based on an entire population with a single-insurer system, which is similar to the overall population of South Korea. Third, the claims database did not include clinical variables such as laboratory values and clinical markers. However, to overcome this limitation, comorbidities were assessed by the CCI score as well as the comorbid disease dummy. Fourth, we estimated the economic burden for the period 2010–2015, since it was the latest available population-based sample data. Further research with more recent data is warranted to examine the economic burden of prescription opioid misuse. Finally, we did not include patients with cancer who might be exposed to the high risk of opioid misuse, and 34% of cancer survivors may have chronic pain which is 20% of the general US population (Dahlhamer et al., 2018; Jiang et al., 2019). Since the focus of this study was to evaluate the opioid misuse in non-cancer patients, we excluded patients with cancer. To understand the burden of opioid misuse in the total Korean population, including patients using opioid with or without cancer, further studies examining the opioid misuse in the patients with cancer would be needed.

## Conclusion

We estimated the incremental direct medical burden of prescription opioid misuse for non-cancer patients using healthcare claims data. Such misuse was significantly associated with an increased economic burden. This result suggested that if we pay attention to opioid misuse, the healthcare expenditure burden can be efficiently managed. Along with appropriate policies to prevent opioid misuse and reduce its economic burden, it is necessary to monitor opioid prescription patterns. Furthermore, the effectiveness of the policies on opioid use should be continuously evaluated.

## Data Availability

The datasets presented in this article are not readily available because the datasets are not accessible and were analyzed only in a remote analysis space provided by the National Health Insurance Service and it is not possible to take out a generated dataset. Requests to access the datasets should be directed to https://nhiss.nhis.or.kr/bd/ay/bdaya001iv.do.
