# Supplementary material for: Economic burden of opioid misuse focused on direct medical costs
Source: Front Pharmacol. 2022 Oct 14;13:928890. doi: 10.3389/fphar.2022.928890 (PMC9620516; doi:10.3389/fphar.2022.928890)
Supplement: Supplementary file 1 [file DataSheet1.docx]

**Appendix A. Top 20 of the major diagnosis for patients with the opioid misuse in terms of the medical cost†**

| Rank | ICD-10 code | Major diagnosis | %‡ |
| --- | --- | --- | --- |
| 1 | M54 | Dorsalgia | 7.75 |
| 2 | M17 | Arthrosis of knee | 7.50 |
| 3 | M48 | Other spondylopathies | 5.97 |
| 4 | I10 | Essential hypertension | 5.33 |
| 5 | M51 | Other intervertebral disc disorders | 5.05 |
| 6 | J20 | Acute bronchitis | 3.47 |
| 7 | M75 | Shoulder lesions | 2.93 |
| 8 | M47 | Spondylosis | 2.78 |
| 9 | M79 | Other soft tissue disorders | 2.42 |
| 10 | E11 | Type 2 diabetes mellitus | 2.42 |
| 11 | N20 | Calculus of kidney and ureter | 2.16 |
| 12 | M50 | Cervical disc disorders | 1.66 |
| 13 | M05 | Seropositive rheumatoid arthritis | 1.57 |
| 14 | M13 | Other arthritis | 1.56 |
| 15 | S33 | Dislocation, sprain and strain of joints and ligaments of lumbar spine and pelvis | 1.24 |
| 16 | J03 | Acute tonsillitis | 1.46 |
| 17 | M19 | Other arthrosis | 1.34 |
| 18 | J06 | Acute upper respiratory infections of multiple and unspecified sites | 1.33 |
| 19 | M45 | Ankylosing spondylitis | 1.31 |
| 20 | N18 | Chronic kidney disease | 0.88 |

**†** The diagnoses for the prescriptions related to opioids

‡ Percentage of the total medical cost

**Appendix B. Top 20 of the major diagnosis for patients with the opioid misuse in terms of the frequency†**

| Rank | ICD-10 code | Major diagnosis | %‡ |
| --- | --- | --- | --- |
| 1 | M54 | Dorsalgia | 11.64 |
| 2 | M17 | Arthrosis of knee | 8.88 |
| 3 | M48 | Other spondylopathies | 5.04 |
| 4 | M51 | Other intervertebral disc disorders | 4.82 |
| 5 | J20 | Acute bronchitis | 4.33 |
| 6 | I10 | Essential hypertension | 4.09 |
| 7 | M79 | Other soft tissue disorders | 3.73 |
| 8 | M75 | Shoulder lesions | 3.43 |
| 9 | M47 | Spondylosis | 3.41 |
| 10 | M13 | Other arthritis | 2.27 |
| 11 | S33 | Dislocation, sprain and strain of joints and ligaments of lumbar spine and pelvis | 2.20 |
| 12 | J06 | Acute upper respiratory infections of multiple and unspecified sites | 1.97 |
| 13 | J03 | Acute tonsillitis | 1.95 |
| 14 | M50 | Cervical disc disorders | 1.44 |
| 15 | E11 | Type 2 diabetes mellitus | 1.39 |
| 16 | M19 | Other arthrosis | 1.26 |
| 17 | M65 | Synovitis and tenosynovitis | 1.23 |
| 18 | J02 | Acute pharyngitis | 1.22 |
| 19 | M25 | Other joint disorders | 1.18 |
| 20 | J00 | Acute nasopharyngitis | 1.05 |
